# The Effect of Bovine Lactoferrin on Fusobacterium nucleatum: A Study on Its Antibacterial and Immunomodulatory Properties in Periodontitis Management

**DOI:** 10.7759/cureus.90279

**Published:** 2025-08-17

**Authors:** Helmi M. A. Khatib

**Affiliations:** 1 Department of Periodontology, Faculty of Dentistry, An-Najah National University, Nablus, PSE

**Keywords:** antibacteria, bovine lactoferrin, fusobacterium nucleatum, in vitro study, periodontitis

## Abstract

Background

Periodontitis is a chronic inflammatory disease that leads to progressive destruction of periodontal tissues, significantly impacting global health. *Fusobacterium nucleatum* (Fn) plays a crucial role in the pathogenesis of periodontitis due to its synergistic interactions with other periodontal pathogens. Lactoferrin (LF), a multifunctional glycoprotein, has shown promising antimicrobial and anti-inflammatory properties, but its effects on Fn have not been fully explored.

Objective

This study aims to assess the antibacterial activity of bovine lactoferrin (bLF) against *F. nucleatum* and to explore its potential as an adjunctive therapy in periodontitis.

Methods

Bovine lactoferrin was tested at various concentrations (2.5, 5, and 10 mg/mL) against *F. nucleatum* in vitro. Antibacterial effects were assessed using optical density (OD) measurements and colony-forming unit (CFU) enumeration. The biocompatibility of bLF was evaluated in L929 fibroblast cells.

Results

Lactoferrin exhibited a dose-dependent inhibition of bacterial growth, with significant suppression of bacterial proliferation at concentrations of 5 and 10 mg/mL (p < 0.05). The antibacterial effects were primarily bacteriostatic, with no complete inhibition observed. Biocompatibility tests indicated that lactoferrin demonstrated acceptable cytocompatibility, with minimal cytotoxicity at lower concentrations.

Conclusions

Bovine lactoferrin shows potential as an adjunctive antimicrobial agent in periodontitis therapy, offering a novel approach to managing *F. nucleatum*-induced periodontal disease. Further in vivo studies are needed to validate these findings and explore their clinical applications.

## Introduction

Periodontitis is a chronic inflammatory disease that causes progressive damage to periodontal tissues, including the gingiva, periodontal ligament, and alveolar bone. It is one of the most prevalent oral diseases globally, affecting nearly 50% of adults [1]. The pathogenesis of periodontitis is primarily driven by a dysbiosis in the subgingival microbiome, where pathogenic bacteria disrupt the balance of the oral microbiota and trigger excessive host immune responses, resulting in tissue destruction [2].

*Fusobacterium nucleatum* (Fn), a Gram-negative anaerobic bacterium, is considered a key periodontal pathogen. It acts as a bridging organism in dental plaque, facilitating the colonization of other bacteria, including *Porphyromonas gingivalis* (Pg), and contributes to periodontal tissue damage through direct cytotoxic effects and the induction of inflammatory cytokines [3].

Current therapeutic strategies for periodontitis primarily involve mechanical debridement (scaling and root planing), often combined with adjunctive antimicrobial agents [4]. However, the rising prevalence of antibiotic-resistant bacterial strains and the risk of microbial dysbiosis caused by broad-spectrum antibiotics highlight the urgent need for alternative treatment options [5,6]. Among these alternatives, lactoferrin (LF), an iron-binding glycoprotein, has demonstrated significant antimicrobial, anti-inflammatory, and immunomodulatory properties, making it a promising candidate for adjunctive periodontal therapy [7,8].

Lactoferrin: Structure, distribution, and antibacterial mechanisms

Structural Characteristics

Lactoferrin (LF) is composed of two homologous lobes (N-lobe and C-lobe), each capable of binding ferric ions with high affinity. This binding is pH-dependent: at acidic pH, LF binds iron tightly, whereas at neutral pH, iron is released. This iron-binding ability is central to its antimicrobial action, as it deprives bacteria of essential iron, a vital nutrient for their growth [9].

Sources and Secretion Pathways

Lactoferrin is secreted by various epithelial cells, including those in the salivary glands, and is also synthesized by neutrophils during immune responses. In the oral cavity, lactoferrin is present in saliva and gingival crevicular fluid, contributing to mucosal defense against pathogens [10].

Antibacterial Mechanisms

Lactoferrin exhibits both bacteriostatic and bactericidal properties.

Iron sequestration: By binding to iron, lactoferrin deprives bacteria of this essential nutrient. This binding is pH-dependent; at acidic pH, such as in periodontal infections, lactoferrin binds iron tightly, whereas at neutral pH, iron is released [11].

Immunomodulation: Lactoferrin modulates the host immune response by stimulating the production of cytokines and chemokines that recruit immune cells to the site of infection. It also enhances the phagocytic activity of immune cells, promoting bacterial clearance [11].

Membrane disruption: The cationic N-terminal region of LF interacts with bacterial membranes, increasing permeability and leading to cell lysis [12].

Antimicrobial peptide generation: Lactoferrin hydrolysis generates lactoferricin and other peptides that target bacterial membranes and lipopolysaccharides (LPS) [13].

## Materials and methods

Materials

Bovine Lactoferrin (bLF)

bLF was obtained commercially and dissolved in sterile distilled water to prepare stock solutions.

Fusobacterium nucleatum (ATCC 25586)

Fn was cultivated in Brain Heart Infusion (BHI) agar and broth under anaerobic conditions at 37°C.

Cell culture

L929 murine fibroblast cells were maintained in Dulbecco's Modified Eagle Medium (DMEM) supplemented with 10% fetal bovine serum (FBS) and 1% penicillin-streptomycin at 37°C in a 5% CO₂ incubator.

Preparation of lactoferrin solutions

Lactoferrin was diluted to concentrations of 10, 5, and 2.5 mg/mL in complete medium. Cells were seeded into 96-well plates and treated with the respective concentrations of lactoferrin for 12 or 24 hours.

Antibacterial assay

Lactoferrin's antibacterial activity was evaluated using a 96-well microdilution assay. Bacterial growth was assessed by measuring the optical density (OD) at 600 nm (OD600), and colony-forming units (CFUs) were enumerated.

Biocompatibility evaluation

The viability of L929 cells was measured using the Cell Counting Kit-8 (CCK-8) assay after treatment with various concentrations of lactoferrin for 12 or 24 hours.

Statistical analysis

Statistical analyses were performed using SPSS version 26 (IBM Corp., Armonk, NY). Data are presented as mean ± standard deviation (SD). Intergroup differences were evaluated using one-way analysis of variance (ANOVA). A statistically significant reduction in bacterial growth was detected at concentrations of 10 and 5 mg/mL compared with the control group (p < 0.05).

## Results

Antibacterial activity of lactoferrin

**Figure 1:**
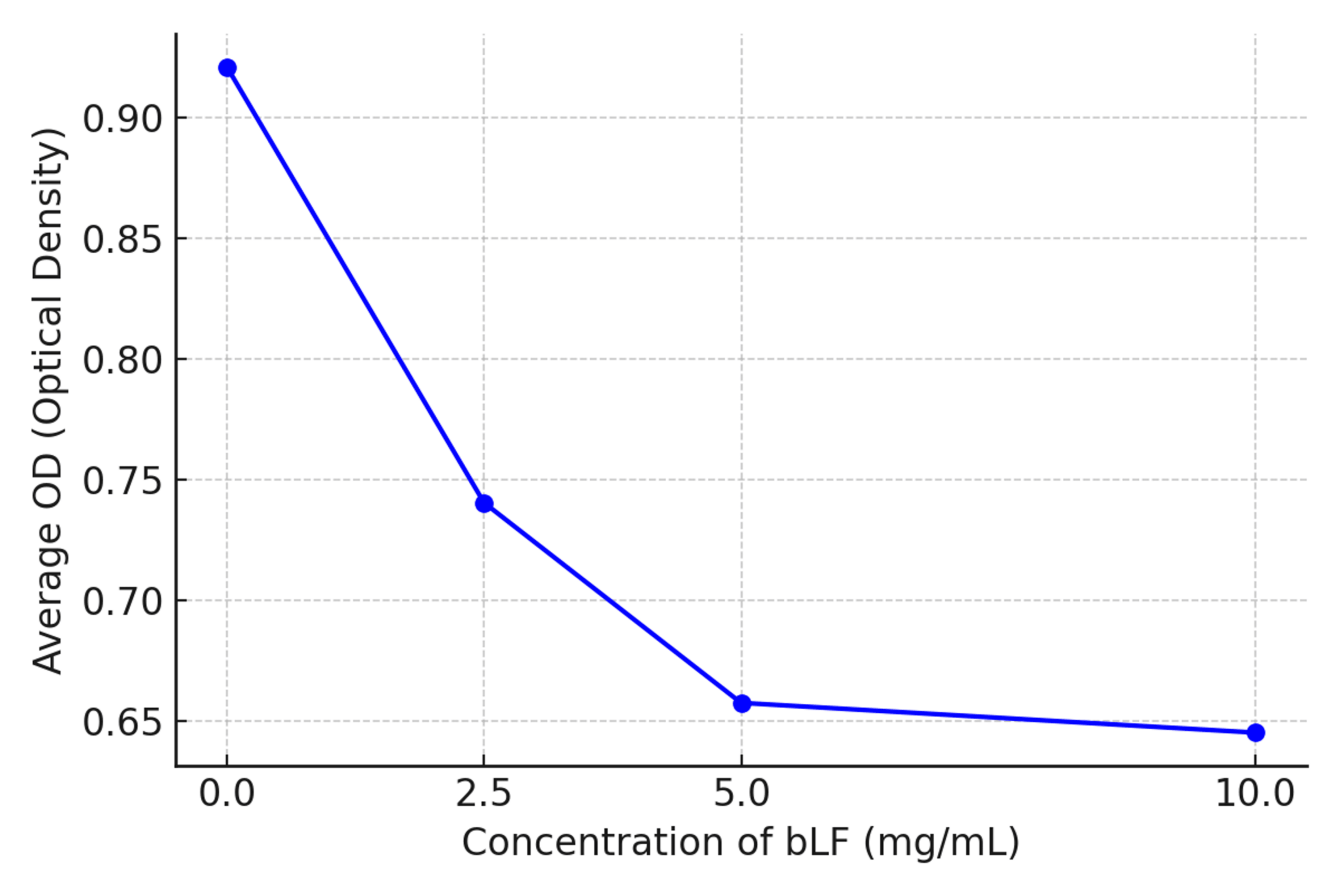
Effect of bLF on the growth of Fusobacterium nucleatum, represented by average optical density (OD600) after treatment with different concentrations of bLF: a dose-dependent inhibition of bacterial growth was observed bLF: bovine lactoferrin, OD: optical density

**Figure 2:**
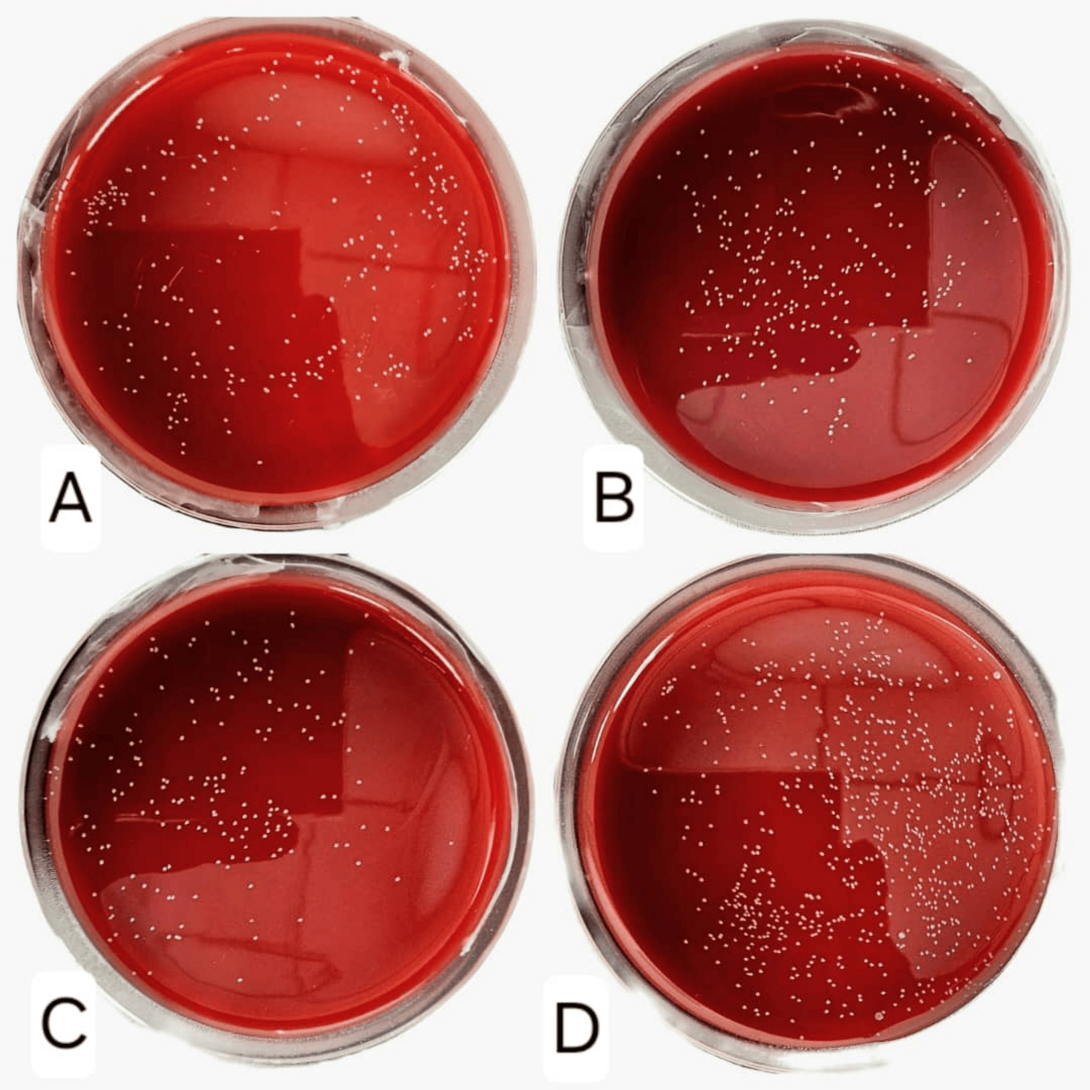
CFUs of Fusobacterium nucleatum after treatment with different concentrations of bovine lactoferrin A: Concentration of bLf is 10 mg/mL. B: Concentration of bLf is 5 mg/mL. C: Concentration of bLf is 2.5 mg/mL. D: Concentration of bLf is 0 mg/mL (control, no lactoferrin added). CFUs: colony-forming units, bLF: bovine lactoferrin

Lactoferrin exhibited a dose-dependent inhibitory effect on the growth of *Fusobacterium nucleatum*. Significant reductions in bacterial growth were observed at concentrations of 10 and 5 mg/mL (p < 0.05), although complete inhibition was not achieved. Optical density measurements at 600 nm (OD600) revealed a decrease in bacterial metabolic activity with increasing concentrations of lactoferrin, indicating a bacteriostatic effect (Figure 1). Furthermore, a corresponding decline in colony-forming units (CFUs) was noted as the concentration of lactoferrin increased, as presented in Figure 2.

Biocompatibility of lactoferrin

**Figure 3:**
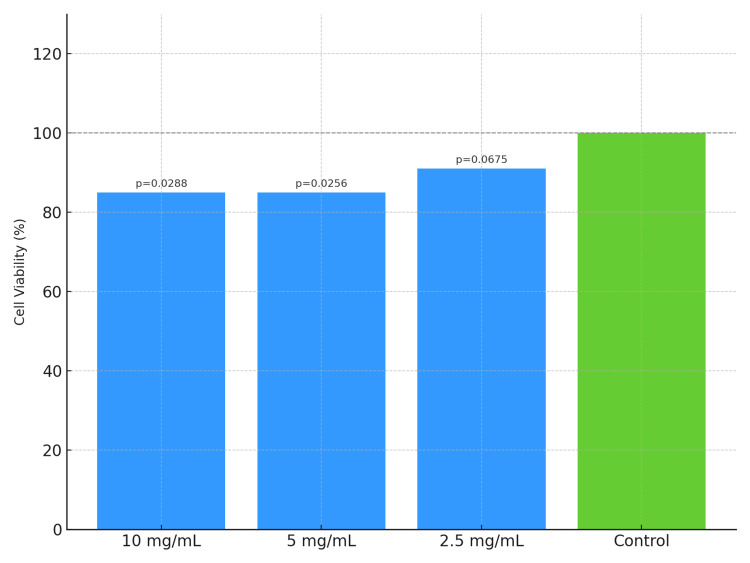
Effect of bovine lactoferrin on L929 cell viability after 12 hours, as assessed by the CCK-8 assay CCK-8: Cell Counting Kit-8

**Figure 4:**
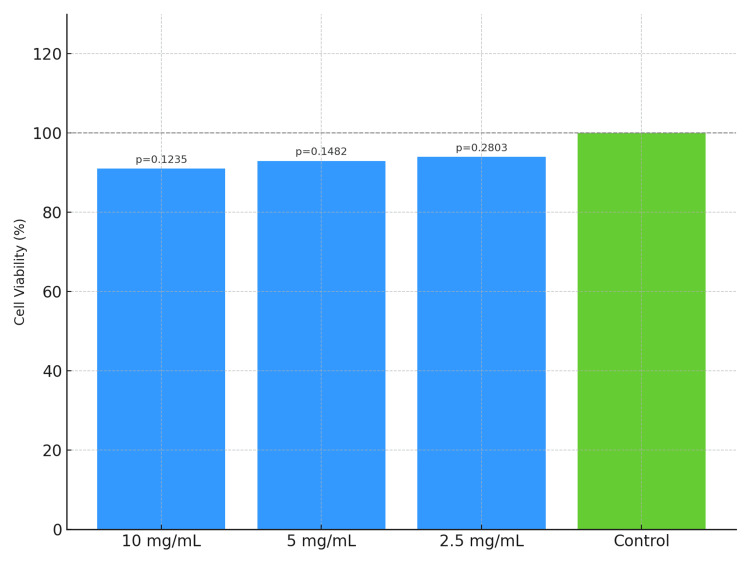
Effect of bovine lactoferrin on L929 cell viability after 24 hours, as assessed by the CCK-8 assay CCK-8: Cell Counting Kit-8

The CCK-8 assay revealed that lactoferrin did not induce significant cytotoxicity in L929 cells at a concentration of 2.5 mg/mL. Higher concentrations (5 and 10 mg/mL) exhibited mild cytotoxic effects. For cell viability after 12 hours of exposure, refer to Figure 3; for results after 24 hours, see Figure 4.

## Discussion

This study investigated the antimicrobial efficacy of bovine lactoferrin (bLF) against *Fusobacterium nucleatum*, a keystone pathogen in the etiology and progression of periodontitis. The findings reveal that bLF exerts a dose-dependent inhibitory effect on *F. nucleatum*, primarily through bacteriostatic rather than bactericidal mechanisms. Although complete bacterial eradication was not achieved under the experimental conditions, the marked suppression of bacterial proliferation highlights bLF's potential utility as an adjunctive therapeutic agent in periodontal disease management.

The antimicrobial properties of lactoferrin can be attributed to multiple mechanisms. Its high-affinity iron-binding capability limits the availability of free iron, an essential cofactor for bacterial metabolic processes, particularly in anaerobic organisms such as *F. nucleatum* [11]. This sequestration of iron is reversible: the addition of iron can restore bacterial growth; therefore, this mechanism is considered bacteriostatic, as it inhibits bacterial proliferation through nutritional immunity rather than directly causing bacterial death.

Beyond iron chelation, lactoferrin also interacts directly with bacterial membranes. The positively charged N-terminal domain of lactoferrin binds to the negatively charged bacterial outer membrane, increasing its permeability and causing leakage of intracellular components, including lipopolysaccharides (LPS). Although this interaction did not result in complete lysis of *F. nucleatum*, it significantly impaired bacterial metabolic activity, as evidenced by the dose-dependent decline in optical density [12].

Furthermore, upon enzymatic cleavage, lactoferrin releases lactoferricin, a cationic peptide derived from its N-terminal region that mediates its iron-independent bactericidal activity. Lactoferricin is several-fold more effective than lactoferrin itself, primarily due to its strong interaction with bacterial membranes. It binds to the lipid A portion of lipopolysaccharides (LPS), disrupting membrane integrity and leading to cell death [13]. Given that *F. nucleatum* is a Gram-negative organism with LPS in its outer membrane, lactoferricin may significantly contribute to the inhibitory effects observed in this study, even in the absence of iron sequestration.

In addition to its direct antibacterial activity, lactoferrin exerts immunomodulatory and anti-inflammatory effects, which are particularly relevant to the pathophysiology of periodontitis. Chronic inflammation is a hallmark of periodontal disease, contributing to tissue destruction and disease progression. Lactoferrin has been shown to interact with immune cells, such as monocytes, dendritic cells, and lymphocytes, modulating cytokine production and attenuating the inflammatory cascade [8]. Thus, lactoferrin's dual functionality, as both an antimicrobial and a host-modulatory agent, positions it as a promising candidate in integrative periodontal therapy.

Nevertheless, the in vitro design of this study imposes certain limitations. The incomplete inhibition of *F. nucleatum* suggests that lactoferrin's effect may be predominantly bacteriostatic under these conditions. While this may be advantageous in preserving beneficial oral microflora, it necessitates further investigation to determine its clinical implications. Moreover, *F. nucleatum* typically exists within complex, multi-species biofilms, which confer increased resistance to antimicrobials. Whether lactoferrin can disrupt or penetrate these biofilms remains unclear and warrants future investigation, particularly given the pivotal role of *F. nucleatum* in biofilm maturation and interspecies aggregation.

Another critical factor is the observed concentration-dependent cytotoxicity. While lower concentrations of bLF (2.5 mg/mL) exhibited minimal adverse effects on fibroblasts, higher concentrations (5 and 10 mg/mL) induced mild cytotoxic responses. This finding emphasizes the need for defining an optimal therapeutic window to ensure efficacy without compromising host cell viability. Local delivery systems, such as gels, rinses, or microspheres, may offer a practical route for achieving effective concentrations at the target site while minimizing systemic exposure.

Looking forward, in vivo studies are essential to validate these findings and assess the therapeutic utility of bLF in periodontal tissues. Animal models of periodontitis may provide critical insights into pharmacodynamics, biofilm interactions, and host response. Moreover, investigating potential synergistic effects between lactoferrin and conventional therapies, such as scaling and root planing or adjunctive antimicrobials, could broaden its clinical applicability. Evaluating lactoferrin's activity against mature biofilms and its impact on microbial community dynamics is also necessary to fully elucidate its potential role in comprehensive periodontal care.

## Conclusions

The present study offers compelling in vitro evidence for the multifaceted therapeutic potential of bovine lactoferrin (bLF) in the context of *Fusobacterium nucleatum*-induced periodontitis. bLF demonstrated a pronounced inhibitory effect on *F. nucleatum* growth, primarily mediated through iron sequestration and disruption of bacterial membrane integrity, indicative of a predominantly bacteriostatic mechanism.

While these findings are promising, translation into clinical application necessitates further investigation. Future research should focus on in vivo validation of bLF efficacy within complex host environments, elucidation of its impact on mature polymicrobial biofilms characteristic of periodontitis, and establishment of optimized dosing protocols that balance therapeutic efficacy with cellular biocompatibility. Notwithstanding these considerations, the dual functionality of bLF, encompassing both antimicrobial and host-modulatory effects, positions it as a strong candidate for development as an adjunctive therapeutic agent. Its potential to complement conventional periodontal treatments and reduce reliance on systemic antibiotics aligns with emerging strategies aimed at precision, host-centered care in the management of periodontal disease.
